# Prediction of Hand Trajectory from Electrocorticography Signals in Primary Motor Cortex

**DOI:** 10.1371/journal.pone.0083534

**Published:** 2013-12-27

**Authors:** Chao Chen, Duk Shin, Hidenori Watanabe, Yasuhiko Nakanishi, Hiroyuki Kambara, Natsue Yoshimura, Atsushi Nambu, Tadashi Isa, Yukio Nishimura, Yasuharu Koike

**Affiliations:** 1 Department of Information Processing, Tokyo Institute of Technology, Yokohama, Japan; 2 Precision and Intelligence Laboratory, Tokyo Institute of Technology, Yokohama, Japan; 3 Department of Developmental Physiology, National Institute for Physiological Sciences, National Institutes of Natural Sciences, Okazaki, Japan; 4 Graduate University for Advanced Studies (SOKENDAI), Hayama, Japan; 5 Precursory Research for Embryonic Science and Technology, Japan Science and Technology Agency, Tokyo, Japan; 6 CREST, Japan Science and Technology Agency, Kawaguchi, Japan; University College of London – Institute of Neurology, United Kingdom

## Abstract

Due to their potential as a control modality in brain-machine interfaces, electrocorticography (ECoG) has received much focus in recent years. Studies using ECoG have come out with success in such endeavors as classification of arm movements and natural grasp types, regression of arm trajectories in two and three dimensions, estimation of muscle activity time series and so on. However, there still remains considerable work to be done before a high performance ECoG-based neural prosthetic can be realized. In this study, we proposed an algorithm to decode hand trajectory from 15 and 32 channel ECoG signals recorded from primary motor cortex (M1) in two primates. To determine the most effective areas for prediction, we applied two electrode selection methods, one based on position relative to the central sulcus (CS) and another based on the electrodes' individual prediction performance. The best coefficients of determination for decoding hand trajectory in the two monkeys were 0.4815±0.0167 and 0.7780±0.0164. Performance results from individual ECoG electrodes showed that those with higher performance were concentrated at the lateral areas and areas close to the CS. The results of prediction according with different numbers of electrodes based on proposed methods were also shown and discussed. These results also suggest that superior decoding performance can be achieved from a group of effective ECoG signals rather than an entire ECoG array.

## Introduction

Over the past two decades, brain-machine interfaces (BMI) have been developed utilizing the growing understanding of brain function and the development of technology to measure brain activity. BMIs translate brain signals into commands for controlling devices such as cursors [1], spelling devices [2], robot arms, and neural prosthetics [3]–[6]. This new communication pathway has not only the potential to help to disabled persons but also provide insight into the motor system of the brain [7]–[13]. A number of methods have been developed to measure brain signals. BMIs are mainly categorized into two types, invasive and non-invasive BMIs, according to the signal source. BMI systems have been developed using modalities such as multi-neuron activity [14], [15], local field potentials [16], [17], electroencephalography [1], [7], [18], [19], and functional magnetic resonance imaging [20].

Electrocorticography (ECoG) has been in focus as a less invasive recording method for BMIs [21]–[36] since the first ECoG-based BMI succeeded in one-dimensional cursor control in human subjects [21]. ECoG signals have higher signal-to-noise ratio and spatiotemporal resolution than non-invasive recording methods, because ECoG electrodes are laid on the surface of the cerebral cortex. ECoG recording has also been shown to have long-term stability [22], [23], and its level of clinical risk is lower compared with invasive methods, because the electrodes do not penetrate the brain. Classifications of arm movement direction [24], [25], 3D cursor control [26], natural grasp type [27], [28], and hand posture [29], [30] have been achieved by using ECoG signals. Two-dimensional [31]–[33] and three-dimensional (3D) hand trajectories [22], [23] and muscle activities [34] have been decoded using epidural or subdural ECoG signals in time series. Despite these successes, however, which locations are most effective for ECoG-based hand trajectory prediction and how different numbers of effective ECoG signals affect decoding performance are still open questions.

In this study, and in investigation of these questions, we attempted to decode hand trajectory from ECoG signals. We recorded 15 and 32 ECoG signals of the primary motor cortex (Ml) and 3D hand positioning simultaneously in two Japanese monkeys while they performed reaching and grasping tasks. We predicted 3D hand trajectories using our previous signal preprocessing method [34] and a partial least squares (PLS) method. Two methods for electrode selection were proposed in order to examine the questions previously mentioned. Prediction performances with different combination of electrodes using the proposed decoding methods were compared. Both methods showed equivalent ability to predict hand trajectories. Our results indicated that 3D hand trajectories can be predicted using nine or ten ECoG signals and that ECoG electrodes with higher performance were concentrated at the lateral areas and areas close to the central sulcus (CS).

## Methods

### Ethics statement

All experimental procedures were performed in accordance with the Guidelines for Proper Conduct of Animal Experiments of the Science Council of Japan and approved by the Committee for Animal Experiments at the National Institutes of Natural Sciences (Approval No.: 11A157). The animals' welfare and steps taken to ameliorate suffering were in accordance with the recommendations of the Weatherall report, “The use of non-human primates in research”. The animals were monitored closely, and their welfare was assessed on a daily basis, or several times a day if necessary. This included veterinary examinations to ensure that they were not suffering, as well as the use of analgesics, antiemetics, or antibiotic therapy if necessary. The animals were housed individually on a 12-hour light/dark cycle and provided a rubber toy and ample food and water in their home cage. No animals were sacrificed in this study.

### Behavioral Task

**Figure 1:**
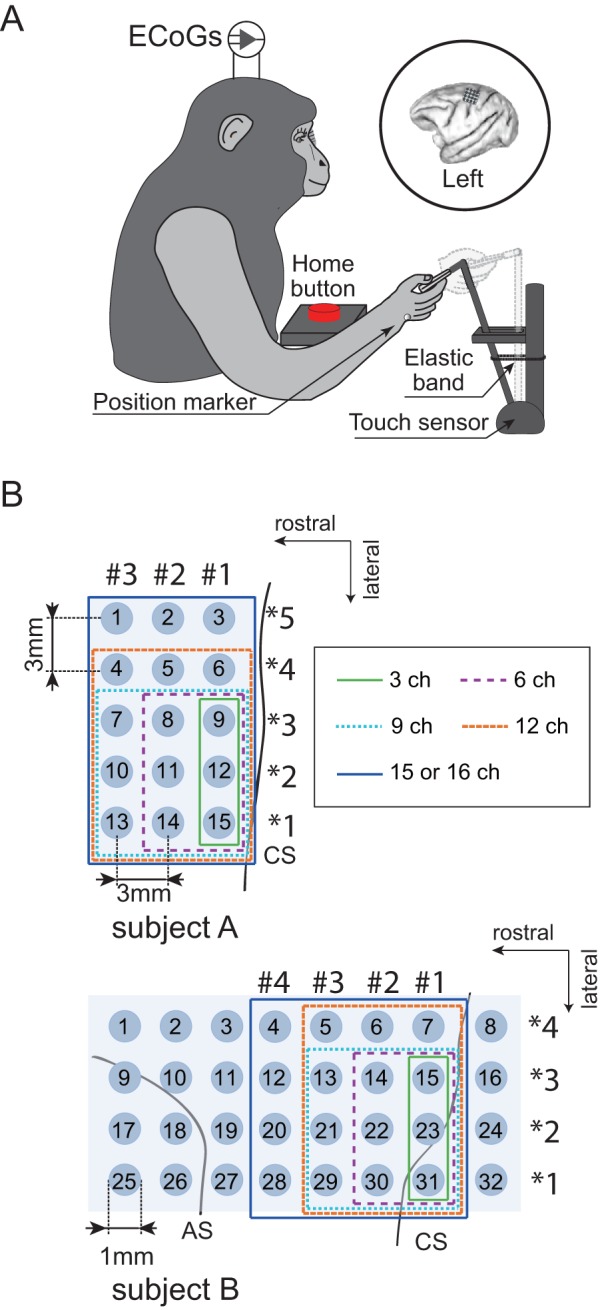
Behavioral task and location of ECoG electrodes used in decoding. A) Monkeys performed right hand reaching, grasping, pulling, and releasing tasks in a 3D workspace. During the task, ECoG and hand positioning were recorded simultaneously. B) The planar-surface platinum electrode arrays were implanted on the gyrus between the central sulcus (CS) and the arcuate sulcus (AS) in the primary motor area in left hemisphere. The locations of all 15 and 32 electrodes in monkey A and monkey B are shown with defined channel numbers. Locations of 3, 6, 9, 12, and 15 or 16 electrode groups used in decoding are denoted with green solid, purple dotted, blue dotted, brown dotted, and blue solid lines, respectively. The column nearest the CS was column #1 in the rostral-caudal direction, and the row in the medial-lateral direction was row *1. Note that electrodes inside the blue line were used in both column and row decoding.

Two Japanese macaques (Monkey A: male, 8.9 kg; Monkey B: female, 4.7 kg) were trained to perform right hand reaching, grasping, pulling, and releasing tasks as shown in Figure 1A. The monkeys performed these tasks repeatedly and continuously for over 700 s. Monkey A performed a total of 134 trials, and monkey B performed 248 trials.

Both monkeys underwent surgery to implant an ECoG electrode array under anesthesia after they completed behavioral training. We chronically implanted a platinum ECoG array (Unique Medical Corporation, Tokyo, Japan) over the left M1, which contained 15 (monkey A: 5×3 grid) and 32 (monkey B: 4×8 grid) channel electrodes, as shown in Figure 1B. Electrode locations were identified from anatomical views during surgery, preparation of brain and postoperative x-ray images. The midline of the brain was estimated from the sagittal suture and used to landmark electrode locations in the medial-lateral direction. The center electrode 8 in monkey A was approximately 15 mm from the midline. The medial electrodes in monkey B were located approximately 14 mm from the midline. The ECoG arrays were nearly parallel to the midline. In the rostral-caudal direction, for both monkeys A and B, centers of electrodes in column #1 (monkey A:3, 6, 9, 12, 15; monkey B: 7, 15, 23) were placed 1–2 mm rostral of the central sulcus. Electrode 31 in monkey B was placed 1–2 mm caudal of the central sulcus. Descriptions of the technical and surgical details can be found in our previous work [34].

### Data recording

ECoG signals were sampled at 4 kHz using an acquisition processor system (Plexon MAP System; Plexon, Inc., Dallas, US). ECoG signals were filtered with band-pass filters through multi-channel bio-signal amplifiers (monkey A: 1.5 Hz high-pass and 1 kHz low-pass analog filters, MEG-6116, Nihon Kohden Corporation, Tokyo, Japan; monkey B: 0.7 Hz high-pass and 8 kHz low-pass analog filters, Plexon, Inc., Dallas, USA).

3D-positions of various points of the right arm were recorded using reflective markers tracked with an optical motion capture system (Eagle Digital System; Motion Analysis Corporation, Santa Rosa, CA). The system used twelve infrared cameras operating at 200 frames/s to track the positions of multiple reflective markers (4-mm-diameter spheroids). A total of fourteen markers were attached to the right arm of each monkey but we used only the wrist marker to extract hand positioning. In addition to optical data, the motion capture system also recorded analog signals from the external stimulator (SEN-8203; Nihon Kohden Corporation, Tokyo, Japan) for synchronization with the neural recordings. The neural data were down-sampled to 500 samples per second, and the motion data were up-sampled to 500 samples per second to match the neural data, similar in manner to our previous work [35].

### Preprocessing and feature selection

Raw ECoG signals were re-referenced to a common average reference (CAR) to increase the signal-to-noise ratio in the preprocessing phase. The CAR method calculates the mean of all channels, and subtracts this value from the selected output channels [36], [37].

**Figure 2:**
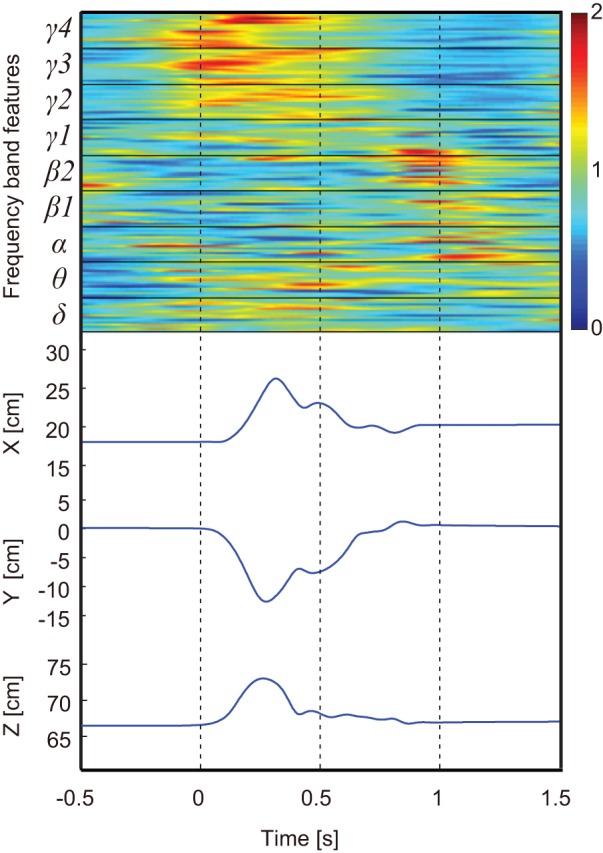
Example of measured trajectory and frequency band feature data during a movement task. Frequency band feature data were sorted into channels and frequency bands, as shown at the top. The X, Y, and Z positioning data recorded from the markers attached to the hand of monkey B, are shown at the bottom.

Nine specific frequency bands were selected for further analysis: δ (1.5∼4 Hz), θ (4∼8 Hz), α (8∼14 Hz), β1 (14∼20 Hz), β2 (20∼30 Hz), γ1 (30∼50 Hz), γ2 (50∼90 Hz), γ3 (90∼120 Hz), and γ4 (120∼150 Hz). These specific bands were selected due to their correlation with motor activity, as shown in previous ECoG-based BMI studies [27]–[34]. Band-pass filters for each of the nine frequency bands were used to transform the re-referenced ECoG signals into nine separate time series. Then, each time series was rectified and smoothed with a Gaussian filter of 0.1 s width (σ: 0.04 s). Finally, the smoothed time series 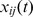
at time *t* were z-score normalized to produce the final ECoG source signal 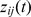
as follows:
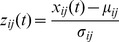
(1)


where, *i* and *j* are the electrode channel and the frequency band, respectively.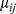 and 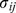 denote the mean value and the standard deviation of 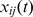 over a 2 s interval before time *t*, respectively. These 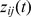 became the final ECoG feature signals for use in hand trajectory prediction. An example ECoG feature signal during a trial movement is shown in Figure 2.

### Partial Least Squares Regression

Partial least squares regression (PLS) was used to decode the 3D hand positioning from ECoG. Because of its utility in variable selection and dimension reduction, PLS has been widely used in the fields of brain imaging, computational chemistry, data mining, and others [22], [23], [38]–[41].

The 3D hand positioning at time *t*, 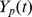, was decoded using the ECoG feature signal 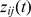 over a 0.6 s interval before time *t* and can be described as 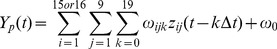
(2)


where, *p* represents the predicted value of each xyz-coordinate, 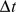 is 30 ms, 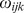 are the weights according to the ECoG feature signal 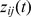 at electrode channel *i*, frequency band *j*, and time 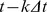, and 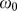 is the bias.

The PLS methods calculates a set of orthogonal factors called latent variables to model the relationship between two sets of data. Ten-fold cross validation was used to evaluate prediction by the model. To avoid over-fitting, the predictive error sum of squares (*PRESS*) was calculated to find the optimal number of latent variables in the PLS model, which can be described as
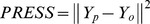
(3)


where 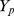
is the predicted hand position, and 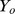
is the observed hand position.

### Two methods for electrode selection

To investigate which electrode locations were more effective, we decreased the number of electrodes for prediction using two methods and compared their respective performance.

In the first method, electrodes were selected based on their implantation position. Previous physiological studies have shown that cortico-motoneuronal cells that encode muscle-activation patterns reflected in EMG activity are located predominantly in the anterior bank of the central sulcus (CS) [42], [43]. Our previous work [34] also showed that the area close to the CS might be key to decoding muscle activity. Therefore, we selected electrodes in groups of 3, 6, 9, 12, and 15 or 16 electrodes, expanding in distance from the CS as shown in Figure 1B. We refer to this method hereafter as location-based selection.

For the second selection method, electrodes were chosen based on prediction performance. Performance values for the PLS model using only one electrode were calculated and sorted by their coefficients of determination (R^2^). Then, electrodes with high performance were added in turn to train a new PLS model. To investigate the effective frequency band for prediction, performance values for the PLS model using only δ (1.5∼4 Hz), γ3 (90∼120 Hz), and γ4 (120∼150 Hz) bands were also calculated for each electrode. We refer to this method hereafter as performance-based selection.

### Analysis

The entire 700 s of experiment data were divided into two parts, 500 s of training data and 200 s of test data. Ten-fold cross validation was employed to train the PLS model on the 500 s of training data. Then, the 200 s of test data were used to evaluate the PLS model.

**Figure 3:**
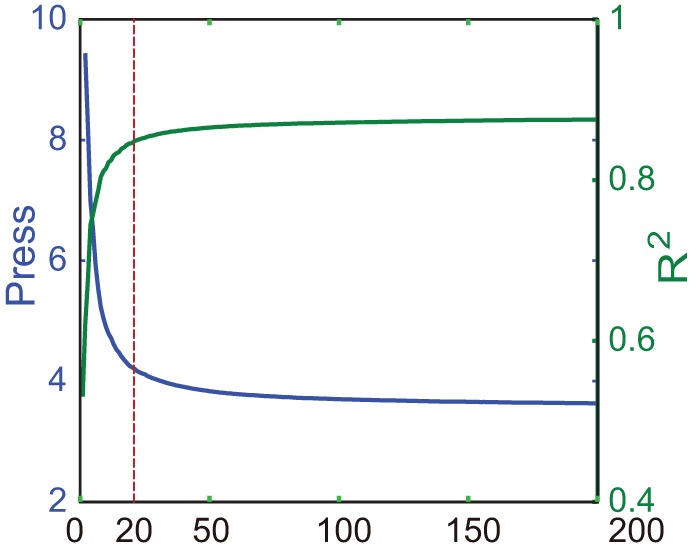
Predictive error sum of squares in model training for monkey B. The blue line and green line show predictive error sum of squares (PRESS) and R^2^ values, respectively, for different numbers of latent variables used in the PLS model. The optimal number of 20 is denoted with the red dotted line.

PRESS values were calculated to find the optimal number of latent variables in the PLS model. Smaller PRESS values were associated with greater PLS model performance. Typically, the PRESS value decreases when effective latent variables are added to train the model. Then, if over-fitting occurs, the PRESS value increases. A good choice is to stop adding latent variables as soon as the PRESS value increases. In this study, however, the PRESS value decreased quickly when the number of latent variables was within 20, but then plateaued soon after, as shown in Figure 3 and Figure S1. Thus, we selected 20 as the optimal number of latent variables.

Weights of the prediction model were analyzed to evaluate the contribution of each the nine frequency bands used in this study. The contribution of frequency band *Con_fb_* was calculated as 
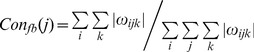
(4)


Where, 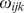 are the weights associated with the ECoG feature signal 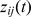 at electrode *i*, frequency band *j*, and time 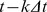.

In addition, 3D hand trajectories were predicted using each of the nine frequency bands of the ECoG feature signals to investigate their individual contributions to prediction.

## Result

### Prediction with the location-based selection method

3D hand trajectories were first decoded using all the ECoG electrodes. For monkey A, the mean R^2^ value and standard deviation (STD) after 10-fold cross validation were 0.4840±0.0118, and mean R^2^ using the test data was 0.4806. For monkey B, the mean R^2^ values after 10-fold cross validation and using the test data were 0.8424±0.0032 (Mean±STD) and 0.7328, respectively.

**Figure 4:**
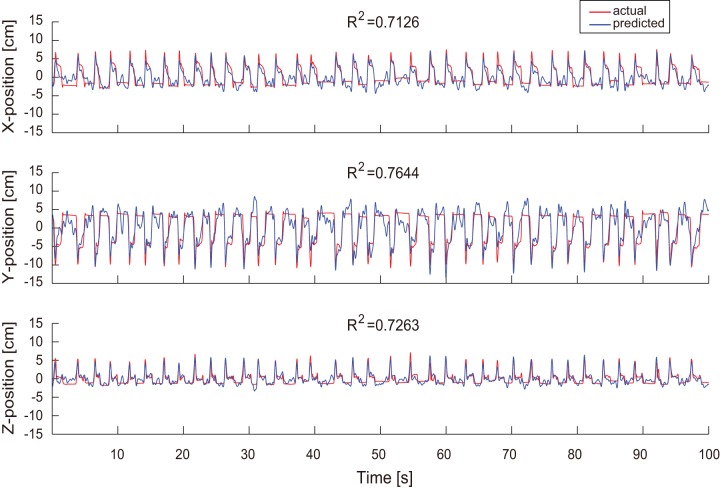
Decoding results using 32 electrodes selected with the location-based method for monkey B. Example of 3D hand trajectory prediction over 100 s of test data using 32 channel ECoG signals. R^2^ values between the predicted (blue) and observed (red) trajectories for X-, Y-, and Z-positions are shown.

**Figure 5:**
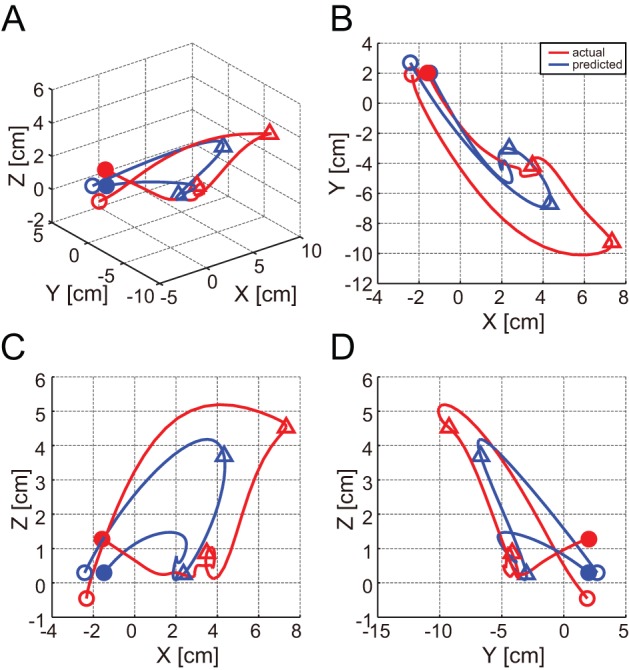
Decoding results for monkey B in three dimensional space. A) Example of 3D hand trajectory prediction for one trial movement using 32 channel ECoG signals. The predicted and observed trajectories in 3D space are depicted in blue and red, respectively. The unfilled circles represent the start point of movement. The two triangles mark hand position at equivalent time points during movement. Solid circles denote the end point of movement. B, C, and D) The predicted (blue) and observed (red) trajectories shown in the X–Y, X–Z, and Y–Z planes, respectively.

We verified how decoding performance changes depending on the number of effective ECoG signals. Positions for the groups of 3, 6, 9, 12, and 15 or 16 ECoG electrodes selected to decode hand trajectories are shown in Figure 1B. For monkey A, R^2^ values for X, Y, and Z positioning were 0.4724, 0.4695, and 0.4997, respectively, obtained using all 15 electrodes. For monkey B with all 32 electrodes, R^2^ values for X, Y, and Z positioning were 0.7126, 0.7644 and 0.7263, respectively. One example of continuous prediction is shown in Figure 4 (see also Fig. S2 for monkey A). Actual and predicted hand trajectories in 3D space for a single trial are also shown in Figure 5 and Figure S3. The best R^2^ values for X, Y, and Z positioning were 0.7288, 0.7677, and 0.7526, respectively, obtained using 9 electrodes of monkey B.

**Figure 6:**
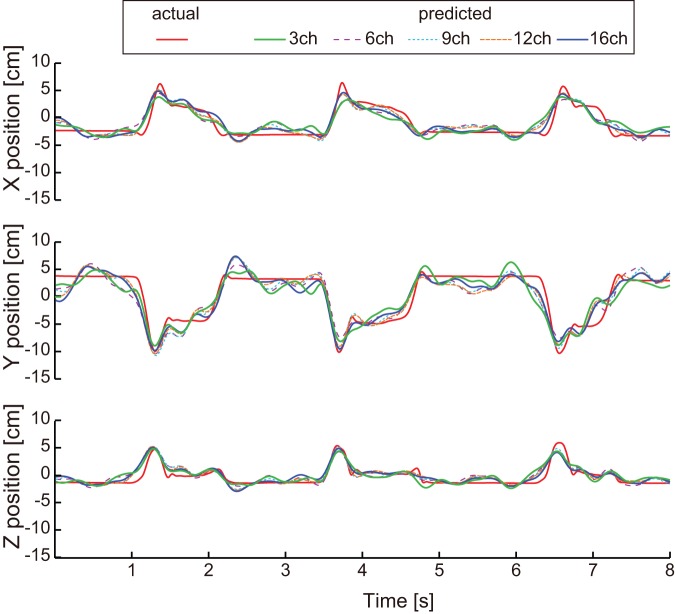
Decoding results for monkey B with different electrode numbers selected using the location-based method. Example prediction of 3D hand positioning over 8 s of test data using 3, 6, 9, 12, and 16 electrodes. The red solid line depicts actual trajectories. The green solid line, purple dotted line, light blue dotted line, brown dotted line, and blue solid line represent predicted trajectories using 3, 6, 9, 12, and 16 electrodes, respectively.

Figure 6 shows prediction results over 8 s of test data using 3, 6, 9, 12, and 16 ECoG electrodes for monkey B (Fig. S4). With the location-based selection method, 67.3% and 92.9% of the best R^2^ values were achieved with 3 electrodes for monkeys A and B, respectively. Best R^2^ percentages using 6 electrodes were 85.3% and 96.9%, and 97.9% and 100% using 9 electrodes.

### Prediction with the performance-based selection method

**Figure 7:**
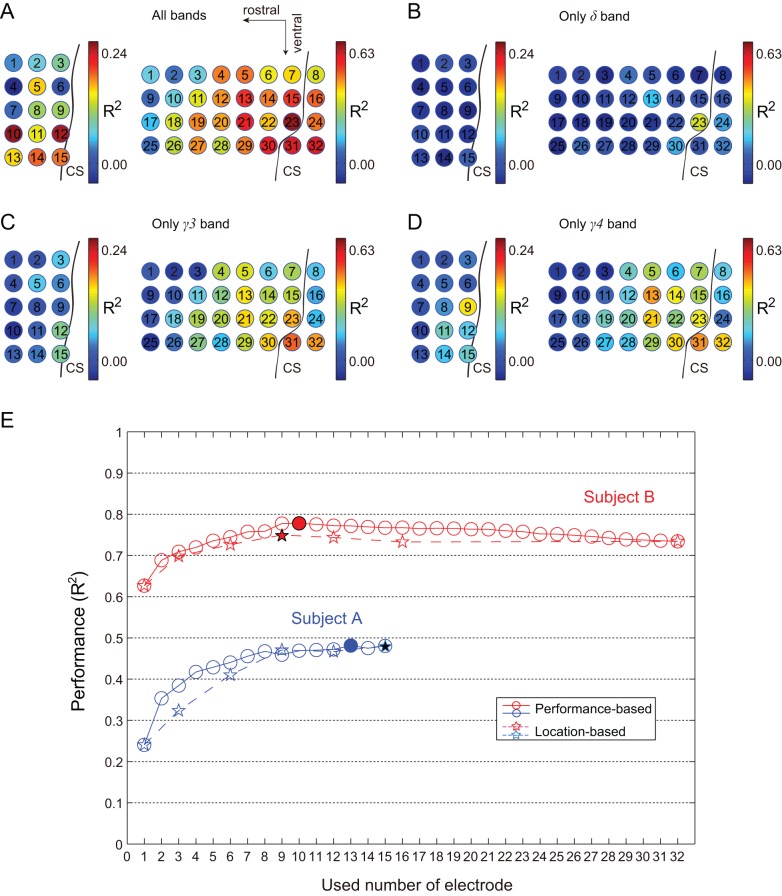
Decoding results for each individual electrode and performance of the two electrode selection methods. A) Prediction performance for each electrode using all frequency bands. The color map in each electrode represents performance of that electrode. R^2^ values ranged from 0.00 to 0.24 for monkey A and 0.00 to 0.63 for monkey B. B) Prediction performance of each electrode using the δ band. For monkey A, R^2^ ranged from 0.00 to 0.24. For monkey B, R^2^ ranged from 0.00 to 0.63. C) Prediction performance of each electrode using the γ3 band. For monkey A, R^2^ ranged from 0.00 to 0.24. For monkey B, R^2^ ranged from 0.00 to 0.63. D) Prediction performance of each electrode using the γ4 band. For monkey A, R^2^ ranged from 0.00 to 0.24. For monkey B, R^2^ ranged from 0.00 to 0.63. E) The blue solid and blue dotted lines represent the decoding performance for monkey A using the performance-based method and location-based method, respectively. The red solid and red dotted lines represent the decoding performance for monkey B using the performance-based method and location-based method, respectively. The solid stars and circles denote the highest performances for the performance-based method and location-based method, respectively.

For the performance-based method, prediction results for each individual electrode are shown in Figure 7A. For monkey A, R^2^ values ranged from 0.0903 to 0.2407. The highest R^2^ value was achieved with electrode 10. For monkey B, R^2^ values ranged from 0.3566 to 0.6269. The highest R^2^ value was achieved with electrode 23. Prediction results for each electrode using δ (1.5∼4 Hz), γ3 (90∼120 Hz), and γ4 (120∼150 Hz) bands are shown in Figure 7B, 7C, 7D, respectively. R^2^ values using the δ band ranged from 0.00 to 0.05 and from −0.06 to 0.37 for monkey A and monkey B, respectively. R^2^ values using the γ3 band ranged from 0.01 to 0.11 and from 0.01 to 0.50, respectively. The R^2^ values using γ4 ranged from 0.01 to 0.16 and from 0.01 to 0.47, respectively. Performances for the γ3 and γ4 bands were similar and generally higher than those of the δ band. For both monkeys, the most effective electrodes were concentrated at the lateral areas and areas close to the CS, especially for the γ3 and γ4 bands.

### Summary of the two electrode selection methods

Performance details of two electrode selection methods are shown in Figure 7E. For both monkeys, performance was improved quickly as the number of electrodes used increased from 1 to 9. The performance curves fluctuated only slightly when using 10 electrodes and above. The best R^2^ values were achieved using 13 and 10 electrodes for monkeys A and B, respectively.

For both methods, the principle is to select more effective electrodes in prediction. As shown in Figure 7A, 7B, 7C, and 7D, higher performance electrodes are concentrated at the lateral areas and near areas of CS. This result is consistent with the principle of the location-based selection method.

To confirm this principle, columns electrodes were also used to predicted hand trajectory. Prediction results in the rostral-caudal direction, and in the medial-lateral direction are shown in Table 1 and Table 2, respectively.

**Table 1:** Prediction results using location-based electrode selection in the rostral-caudal direction.

Monkey	Location	R^2^			
		x	y	z	mean
A	#1	0.3317	0.3397	0.3535	**0.3416**
	#2	0.2959	0.2452	0.3438	0.2950
	#3	0.2998	0.2956	0.3580	0.3178
B	#1	0.6773	0.7164	0.6980	**0.6973**
	#2	0.6063	0.6593	0.6524	0.6393
	#3	0.6591	0.6793	0.6704	0.6696
	#4	0.5655	0.6059	0.5578	0.5763

**Table 2:** Prediction results using location-based electrode selection in the medial-lateral direction.

Monkey	Location	R^2^			
		x	y	z	mean
A	*1	0.3035	0.3008	0.3429	0.3157
	*2	0.3640	0.3774	0.4141	**0.3852**
	*3	0.2423	0.2217	0.2873	0.2505
	*4	0.1996	0.1807	0.2381	0.2061
	*5	0.1851	0.1713	0.2213	0.1926
B	*1	0.6343	0.6888	0.6117	0.6449
	*2	0.6577	0.6930	0.7069	**0.6859**
	*3	0.6646	0.7005	0.6841	0.6830
	*4	0.5729	0.5865	0.5501	0.5699

The highest performance in the rostral-caudal direction was achieved using column #1 in both monkeys. For monkey A, performance using column #3 was higher than that using #2. This might have been an effect of the presence of electrode 10 (Figure 7A). For monkey B, performance using column #3 was second highest, and performance using column #2 was higher than that using column #4. Highest performance in the medial-lateral direction was achieved using row *2 in both monkeys. For monkey A, performance using row *1 was higher than that using rows *3, *4, and *5. For monkey B, performance using row *3 was second highest, and may have been due to the effect of the δ band at electrode 13 (Figure 7B). Performance using row *1 was higher than that using row *4. Generally, higher performance rows and columns are at the lateral areas and areas near the CS.

### Analysis of specific frequency bands

**Figure 8:**
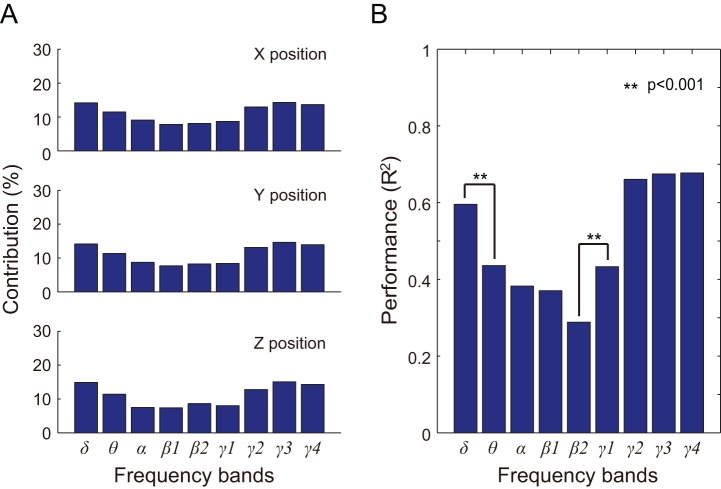
Contribution and performance of specific frequency bands for monkey B. A) Each bar represents the weight of each frequency band in the PLS model. From top to bottom, the graphs depict contributions to X, Y, and Z positioning, respectively. B) Prediction results using each frequency band. We performed a two-way ANOVA with effects positioning and frequency bands. Each bar represents the mean R^2^ value of X, Y, and Z positioning when comparing observed and predicted hand trajectories. Significant differences between mean R^2^ values are denoted with * (p<0.001).

Weights of the nine frequency bands in the prediction model were calculated and are shown in Figure 8A as percent contributions. For monkey B, γ3 (90∼120 Hz) provided the highest contribution. The contributions of δ (1.5∼4 Hz) and γ4 (120∼150 Hz) were higher than those of θ (4∼8 Hz), α (8∼14 Hz), β1 (14∼20 Hz), and β2 (14∼20 Hz).

3D hand trajectories were predicted by using each frequency band of the ECoG feature signals individually (Table 3). A two-way ANOVA was employed to judge two effects (X, Y, and Z positioning, and the nine frequency bands). No significant differences in prediction performance between X, Y, and Z positioning were observed in both monkeys (monkey A: *F*_2, 16_  = 3.61, *p*  = 0.051; monkey B: *F*_2, 16_  = 1.96, *p*  = 0.173). Significant differences in prediction performance were observed between frequency bands, (monkey A: *F*_8, 16_  = 14.16, *p*  = 6.41×10^−6^; monkey B: *F*_8, 16_  = 52.39, *s*  = 4.99×10^−10^), as shown in Figure 8B. The prediction performances using δ, γ2, γ3, and γ4 bands were also significantly higher than that of other bands. Prediction performance of the θ and γ1 bands was significantly higher than that of β2.

**Table 3:** Prediction results using individual frequency bands.

Monkey	Bands	R^2^			
		x	y	z	mean
A	δ	0.0987	0.0440	0.1121	0.0850
	θ	0.0689	0.0873	0.0757	0.0773
	α	0.0793	0.1094	0.0800	0.0895
	β1	0.1314	0.1206	0.1503	0.1341
	β2	0.1860	0.2108	0.2188	0.2052
	γ1	0.1627	0.1808	0.1634	0.1690
	γ2	0.1604	0.1476	0.2033	0.1705
	γ3	0.1602	0.1149	0.1920	0.1557
	γ4	0.1739	0.1584	0.2103	0.1809
B	δ	0.5652	0.5970	0.6242	0.5955
	θ	0.4316	0.4496	0.4263	0.4358
	α	0.4169	0.4252	0.3051	0.3824
	β1	0.3743	0.4049	0.3318	0.3703
	β2	0.2906	0.3389	0.2345	0.2880
	γ1	0.4567	0.4796	0.3618	0.4327
	γ2	0.6391	0.6794	0.6628	0.6605
	γ3	0.6427	0.6844	0.6966	0.6746
	γ4	0.6477	0.6913	0.6929	0.6773

## Discussion

This study decoded 3D hand trajectories from ECoG signals in Ml and showed that most effective electrodes were concentrated at the lateral areas and areas close to the CS. Comparisons between prediction results suggest that a selection of effective ECoG signals may be better choice than a whole ECoG array. Our results also suggested that ECoG signals are of ample quality and efficiency to control a high performance neural prosthetic.

### Which locations are most effective for prediction?

Carmena et. al. (2003) reported that neuron activity recorded from Ml showed greater efficacy than that from dorsal premotor cortex, supplementary motor cortex, posterior parietal cortex, and primary somatosensory cortex. Previous ECoG studies have also used signals mainly from the primary motor area [27]–[29]. We chose M1 based on those previous results and evaluated the optimal locations in M1. As shown in Figure 7A, ECoG signals from the lateral areas and near areas of CS also showed greater efficacy in prediction, especially in the δ, γ3, and γ4 bands (Figure 7B, 7C, 7D).

### How did different numbers of ECoG electrodes affect performance?

As shown in in Figure 7E, the best mean R^2^ values for monkeys A and B were 0.4805 and 0.7496, respectively, in the location-based selection, and 0.4815 and 0.7780 in the performance-based selection. Both methods, therefore, appear to have equivalent ability to predict hand trajectories.

For both monkeys, performance improved quickly as the number of electrodes used increased from 1 to 9. The performance curves fluctuated only slightly when using 10 electrodes and above. Best decoding performance was achieved using a relatively small number of electrodes, 13 and 10 electrodes in the performance-based selection for monkey A and monkey B, respectively. The performances curves of this study are similar to the results of a previous neuron activity-based study [14], which selected different numbers of high sensitivity neurons in decoding kinematic variables. These results suggest that best decoding performance can be achieved from a relatively small number of effective ECoG signals. However, it should also be noted that decoding performance is not simply related to the electrode number but may more closely depend on the density of electrodes within the effective areas. Still, with the potential utility of wireless transmission technology in ECoG [44], [45], a relatively smaller number of electrodes would provide the benefit of lower power consumption, extending the usage time for wireless BMIs.

### Which frequency bands are most effective?

To evaluate the efficacy of specific frequency bands in trajectory decoding, we compared prediction performances of the nine physiologically-based frequency bands with 10 Hz-width fractionized frequency bands from 0 to 150 Hz. The physiologically-based method produced nearly the same or better results (R^2^  = 0.7328) than the fractionized frequency method (R^2^  = 0.6815) for monkey B. These results suggest that the usage of physiological frequency bands is more effective than non-physiological fractionized frequency bands.

Weight analysis for the PLS model and the results of decoding performance using each of the nine frequency bands showed that the δ, γ2, γ3, and γ4 bands were more effective than other bands in this study. Previous ECoG studies have shown the importance of the high γ band in motor decoding and BMI control, such as the 60–80 Hz band in prediction of 3D hand trajectories in monkeys [22], [23], 70–110 Hz in controlling a 3D cursor in humans [26], and 56–128 Hz in grasp detection in humans [28]. The importance of the δ band is also supported by our previous ECoG work [34], and is consistent with a previous study [27], which employed a low-frequency band (2–6 Hz) to classify natural grasp types.

## Supporting Information

Figure S1 **Predictive error sum of squares in model training for monkey A.** The blue line and green line show predictive error sum of squares (PRESS) and R2 values, respectively, for different numbers of latent variables used in the PLS model. The optimal number of 20 is denoted with the red dotted line.
(EPS)

Figure S2 **Decoding results using 15 electrodes for monkey A.** Example of prediction of 3D hand positions during 100 seconds test data by using 15 channel ECoG signals. The R2 value between the predicted (blue) and observed (red) trajectories for X-, Y-, and Z-positions are shown.
(EPS)

Figure S3 **Decoding results for monkey A in three dimensional space.** A) Example of 3D hand trajectory prediction for one trial movement using 3 electrodes. The predicted and observed trajectories in 3D space are depicted in blue and red, respectively. The unfilled circles represent the start point of movement. The two triangles mark hand position at equivalent time points during movement. Solid circles denote the end point of movement. B, C, and D) The predicted (blue) and observed (red) trajectories shown in the X–Y, X–Z, and Y–Z planes, respectively.
(EPS)

Figure S4 **Decoding results for monkey A with different electrode numbers selected using the location-based method.** Example prediction of 3D hand positioning over 8 s of test data using 3, 6, 9, 12, and 15 electrodes. The red solid line depicts actual trajectories. The green solid line, purple dotted line, light blue dotted line, brown dotted line, and blue solid line represent predicted trajectories using 3, 6, 9, 12, and 15 electrodes, respectively.
(EPS)
